# Risk prediction models for oral clefts allowing for phenotypic heterogeneity

**DOI:** 10.3389/fgene.2015.00264

**Published:** 2015-08-13

**Authors:** Yalu Wen, Qing Lu

**Affiliations:** ^1^Department of Statistics, University of Auckland, AucklandNew Zealand; ^2^Department of Epidemiology and Biostatistics, Michigan State University, East Lansing, MIUSA

**Keywords:** sub-phenotype, multi-class likelihood-ratio ensemble method, cleft lip with or without palate, cleft palate only, genetic heterogeneity

## Abstract

Oral clefts are common birth defects that have a major impact on the affected individual, their family and society. World-wide, the incidence of oral clefts is 1/700 live births, making them the most common craniofacial birth defects. The successful prediction of oral clefts may help identify sub-population at high risk, and promote new diagnostic and therapeutic strategies. Nevertheless, developing a clinically useful oral clefts risk prediction model remains a great challenge. Compelling evidences suggest the etiologies of oral clefts are highly heterogeneous, and the development of a risk prediction model with consideration of phenotypic heterogeneity may potentially improve the accuracy of a risk prediction model. In this study, we applied a previously developed statistical method to investigate the risk prediction on sub-phenotypes of oral clefts. Our results suggested subtypes of cleft lip (CL) and palate have similar genetic etiologies (AUC = 0.572) with subtypes of CL only (AUC = 0.589), while the subtypes of cleft palate only (CPO) have heterogeneous underlying mechanisms (AUCs for soft CPO and hard CPO are 0.617 and 0.623, respectively). This highlighted the potential that the hard and soft forms of CPO have their own mechanisms despite sharing some of the genetic risk factors. Comparing with conventional methods for risk prediction modeling, our method considers phenotypic heterogeneity of a disease, which potentially improves the accuracy for predicting each sub-phenotype of oral clefts.

## Introduction

Oral clefts comprise a significant component of birth defects. Individuals born with orofacial clefts are likely to require subsequent dental, speech, and psychosocial therapies to correct for the craniofacial anomalies to various degrees (Strauss, 1999; Mossey et al., 2009; Wehby and Cassell, 2010). Traditionally, oral clefts are classified into cleft palate only (CPO) and cleft lip (CL) with or without palate (CL/P; Fraser, 1955, 1970). The birth prevalence of CL/P is about 1/700 with wide variability associated with geographic origin, whereas CPO affects 1/2500 births with less variability compared to CL/P (Mossey, 2003; Genisca et al., 2009; Beaty et al., 2011; Dixon et al., 2011). Oral clefts can also be divided into non-syndromic and syndromic forms, where approximately 70% of CL/P and 50% of CPO are non-syndromic (Jones, 1988; Stoll et al., 2000; Calzolari et al., 2007; Genisca et al., 2009; Jugessur et al., 2009; Dixon et al., 2011). With the advent of genomic era, major breakthroughs have been made into identifying genetic variants predisposing to the syndromic oral clefts, while our knowledge of non-syndromic oral clefts genetic etiology were still far behind. This could be partially due to the heterogeneous etiology and the non-Mendelian inheritance pattern of non-syndromic oral clefts (Murray, 2002; Dixon et al., 2011). For this particular reason, for the rest of this paper we focus on non-syndromic oral clefts with the consideration of their phenotypic heterogeneity.

Compelling evidences suggest that genetic variants play a substantial role in the development of oral clefts (Little and Bryan, 1986; Wyszynski et al., 1998; Murray, 2002; Mossey et al., 2009; Grosen et al., 2010; Beaty et al., 2011; Grosen et al., 2011). Twin studies indicate that the concordance rates of CL, cleft lip and palate (CLP), and CPO are higher for monozygotic twins than for dizygotic twins (Little and Bryan, 1986; Murray, 2002; Grosen et al., 2010, 2011; Beaty et al., 2011). For example, in a nationwide study in Denmark, the proband-wise concordance rate is 33% for monozygotic twins while the rate is 7% for dizygotic twins, which is only slightly higher than the 3% recurrence risk observed for full siblings (Grosen et al., 2010, 2011). Moreover, the recurrence risk of oral clefts in families is greater than that predicted by the familial aggregation of environmental risk factors. Evidence also shows that the risk of oral clefts among first degree relatives of cases is much higher than that in the general population (Wyszynski et al., 1998; Sivertsen et al., 2008). Conventionally, CL/P and CPO are treated separately, because the developmental origins of these two defects are different during the embryonic stage. Through genetic linkage studies, various loci and genetic regions, such as *MTHFR*, *TGFA*, and *TGFB3*, were found to play a potential role in CL/P (Prescott et al., 2000; Zeiger et al., 2003). Genes related to growth factors [e.g., *TGFA* (Mitchell, 1997; Zeiger et al., 2005; Vieira, 2006)], transcription factors [e.g., *IRF6*(Zucchero et al., 2004; Park et al., 2007; Vieira et al., 2007a,b; Jugessur et al., 2008)], nutrient metabolism [e.g., *MTHFR* (Vieira et al., 2005)], and immune response [e.g., *PVRL1*(Sozen et al., 2001)] have also been examined through genetic association studies. As with many other candidate gene studies, rigorous confirmatory replication is not common, except for the gene *IRF6*, which is linked strongly to the isolated form of clefts. The association finding of *IRF6* with CL/P has been replicated in many different populations and ethnic groups (Zucchero et al., 2004; Park et al., 2007; Vieira et al., 2007a,b; Jugessur et al., 2008; Mossey et al., 2009). To date, much of the attention has been paid to CL/P rather than CPO among non-syndromic oral clefts (Beaty et al., 2011; Dixon et al., 2011). This may be explained by relatively large samples of CL/P that are available, better ascertainment and less confounding issues for CL/P as compared to CPO (Dixon et al., 2011). More studies will be needed to fill in the gap to shine light on the underlying biological mechanism of CPO. Despite these discoveries, the results from both linkage and association studies are largely inconsistent, indicating the challenge of identifying disease-associated genetic variants for complex diseases with heterogeneous etiology (Carter et al., 1982; Harville et al., 2005; Sivertsen et al., 2008; Mossey et al., 2009; Dixon et al., 2011).

With the increasing genetic and epidemiologic findings for oral clefts, the translation of these discoveries into health practice becomes one of the major challenges of the coming decades. It is hoped that the genetic risk prediction could help identify sub-population at high risk of oral clefts and then advanced disease prevention and intervention strategies can be used to reduce the risk. Despite such promise, the existing genetic findings are insufficient to explain the familial aggregation of oral clefts (Mossey et al., 2009; Dixon et al., 2011) and as the result the risk prediction models for oral clefts formed to date have lacked sufficient accuracy for clinical use. Part of this difficulty is due to the phenotypic heterogeneity, i.e., oral clefts with the same or similar clinical manifestations have different genetic etiologies. When heterogeneous sub-phenotypes were treated as a single entity, the predictive power of the disease-associated variants could be substantially reduced, leading to a prediction model with low accuracy (Morris et al., 2010; Dixon et al., 2011). The use of more refined sub-phenotypes defined based upon disease symptom, severity of illness, and age at onset, facilitates the identification of new genetic variants contributing to each sub-phenotype, and helps build a more accurate risk prediction model (Morris et al., 2010). The improved risk prediction model could be used to identify high risk sub-population deserving special attention so that more targeted prevention and intervention strategies can be used to reduce the mortality and morbidity. However, in the absence of a well-defined diagnosis criterion to classify oral clefts into more homogeneous sub-phenotype groups, a risk prediction model simply built on each sub-phenotype could be subject to low accuracy and high variability because of the small sample size for each sub-phenotype. Recently, a multi-class likelihood-ratio ensemble (MLRE) method has been proposed. It gradually combines sub-phenotype groups with similar genetic etiology into homogeneous subgroups, and in general does not require any prior knowledge of subgroup information (Wen and Lu, 2013). With explicitly accounting for phenotypic heterogeneity, the method has been shown to have greater power over the existing methods. By applying the new method to a large-scale oral clefts genetic data, we simultaneously consider nine refined sub-phenotypes of non-syndromic oral clefts defined primary based on clinical manifestations, and gradually combine sub-phenotypes with similar genetic etiology. We further build risk prediction models on each combined sub-phenotype group by considering 148 candidate single nucleotide polymorphisms (SNPs) and their potential interactions.

## Materials and Methods

### The International Consortium to Identify Genes and Interactions Controlling Oral Clefts (ICOCs) Study Dataset

The Interactions Controlling Oral Clefts (ICOCs) is one of the largest and most comprehensive family based studies conducted to date, aimed at discovering genes and interactions contributing to oral clefts. It was developed through the trans-*NIH* Genes, Environment, and Health Initiative (*GEI*) and pulled together a large collection of cases and their parents from multiple populations based on similar research protocols (Beaty et al., 2010; Cornelis et al., 2010), which comprised nearly 1908 case-parent trios from different racial groups, including Caucasian, African Americans, and Asians. DNA samples were collected from both cases and their parents, and were genotyped using the Illumina Human 1M DNA Analysis Bead Chip. Cases were affected offspring identified through a treatment center of population based registry and were individuals diagnosed with an isolated, non-syndromic oral cleft that included soft CPO, hard CPO, left cleft lip, right cleft lip, bilateral cleft lip, left cleft lip, and palate, right cleft lip, and palate, bilateral cleft lip, and palate, microforms of oral cleft, and unknown types of oral clefts. Parents of the affected children were also recruited for the study. The DNA samples were genotyped with Illumina’s 610 Quad platform at the Johns Hopkins University Center for Inherited Disease Research (*CIDR*). Based on existing literature, we have identified 148 SNPs potentially associated with oral cleft, which were available in the dataset. Due to the small sample size, individuals with microforms and bilateral cleft lip were excluded from the analyses. Individuals with unknown types of oral clefts were also excluded from the analyses, as the genetic causes for unknown types of oral clefts could be highly heterogeneous.

### Genetic Risk Prediction Analysis Allowing for Phenotypic Heterogeneity

In this risk prediction analysis, we use a newly developed MLRE, method (Wen and Lu, 2013) to take the heterogeneous nature of oral clefts into account. The MLRE method gradually combines sub-phenotypes into homogeneous groups based on the genetic similarities among sub-phenotypes. It assumes no mode of inheritance and allows for the identification of high-order gene-gene interactions by using a computationally efficient forward selection algorithm. The MLRE starts with all sub-phenotype groups under investigation and treats each sub-phenotype as a distinct disease outcome. It then applies the receiver operating characteristic (ROC)-based forward selection algorithm to identify genetic risk factors for each sub-phenotype and then measures the pair-wise genetic similarities among all sub-phenotypes. It gradually combines the most similar sub-phenotypes and evaluates the overall classification accuracy of the model using a global ROC statistic, which addresses both the lack of accuracy due to the presence of heterogeneity among sub-phenotypes and the accuracy loss caused by reduced sample size when some sub-phenotypes indeed share the same genetic etiology. The process continues until all sub-phenotypes are clustered into one group. The optimum number of sub-phenotype groups with sufficient sample size of each group and least possible phenotype heterogeneity is determined through a *K*-fold cross validation procedure using the global ROC-statistic as a criterion. With the selected optimal number of sub-phenotypes, the method is then applied to all samples to build a risk prediction model for each sub-phenotype group. Through extensive simulation studies, Wen and Lu (2013) have demonstrated that MLRE attained higher accuracy compared with commonly adopted methods under various underlying numbers of sub-phenotype groups and disease models.

The major advantage of MLRE over existing methods (e.g., those analyzing sub-phenotype separately or analyzing all sub-phenotypes as a single entity) is that MLRE incorporates genetic information obtained from data and gradually combines sub-phenotype groups that share similar genetic etiology to improve both accuracy and precision of risk prediction models. Therefore, it overcomes the obstacles when the prior information of defining sub-phenotypes is lacking or inaccurate, and provides a powerful and flexible framework to search for genetic variants contributing to complex human diseases, allowing for heterogeneous genetic causes among sub-phenotypes of a disease.

## Results

### Descriptive Analysis

**Table 1** describes the samples of all sub-phenotypes and controls in relation to the distribution of gender in the ICOC dataset. Noteworthy, while the distribution of gender among controls is roughly balanced, there is an excess of cleft palate cases in females and an excess of CL with/without palate cases in males.

**Table 1:** Distribution of sub-phenotypes in the Interactions Controlling Oral Clefts (ICOCs) dataset.

Sub-phenotypes of oral clefts		All	Male	Female
Control		3692	1759	1933

Cleft palate only	CP-Soft only	223	85	138
	CP- Hard	172	76	96

Cleft lip only	Left CL	244	152	92
	Right CL	114	66	48
	Bilateral CL	30	15	15

Cleft lip and palate	Left CLP	485	314	171
	Right CLP	271	183	88
	Bilateral CLP	336	240	96

Unknown		615	361	254

Microforms		3	2	1

### Risk Prediction Modeling

**FIGURE 1:**
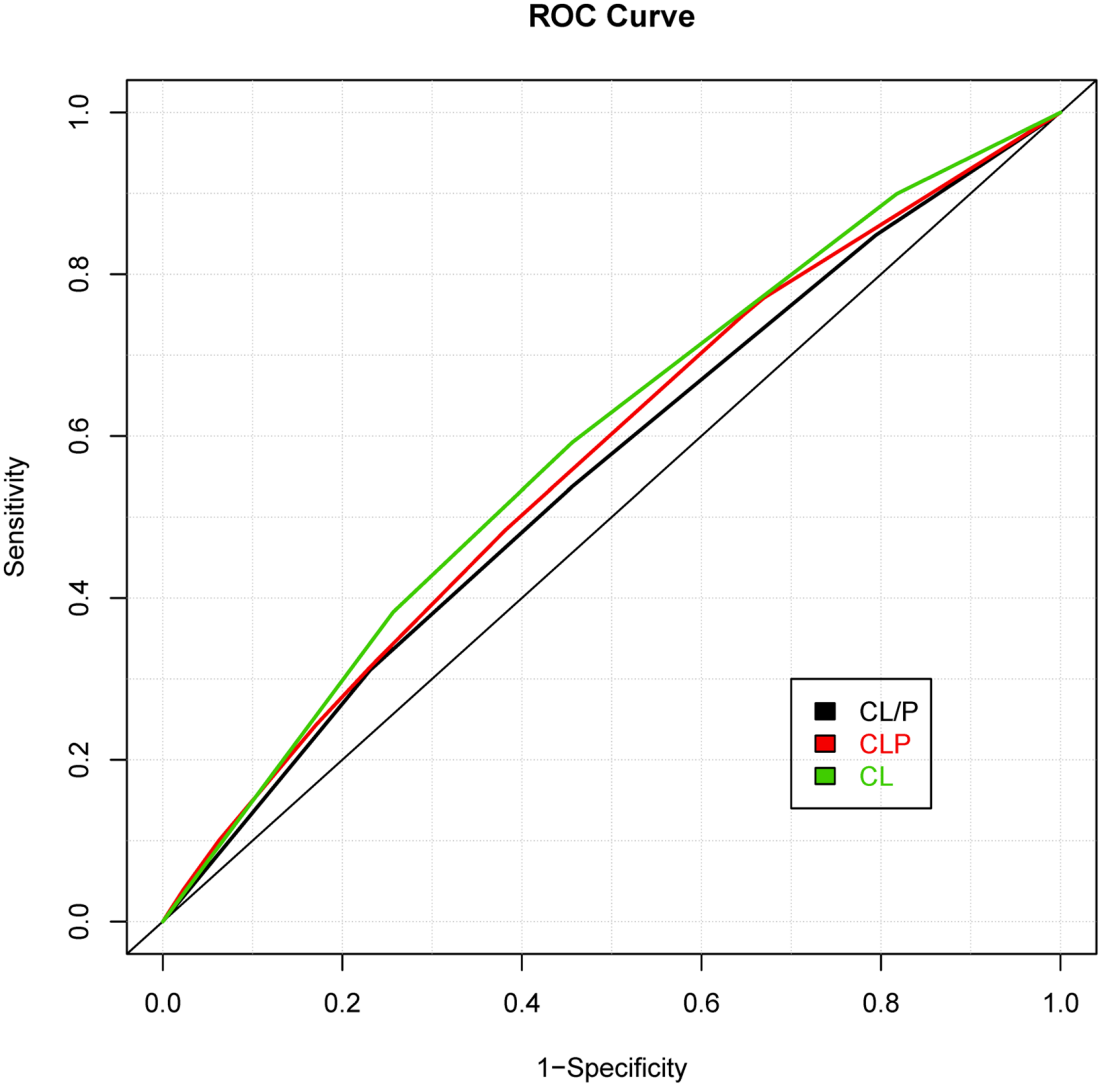
**Receiver operating characteristic (ROC) curves of risk prediction models for cleft lip with and without palate (CL/P), cleft lip with palate (CLP), and cleft lip only (CL)**.

**FIGURE 2:**
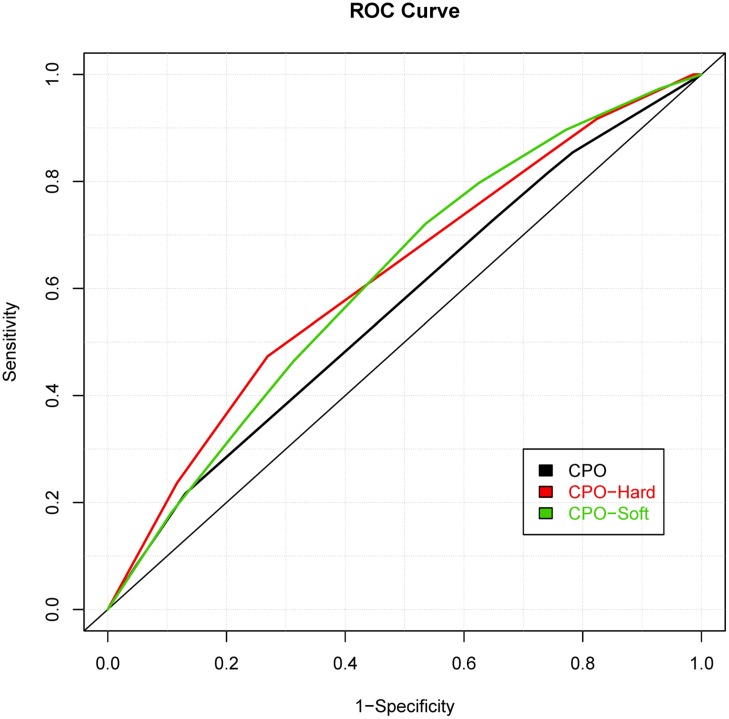
**Receiver operating characteristic curves of risk prediction models for cleft palate only (CPO), cleft palate-hard and cleft palate-soft**.

While prior studies have focused on building risk prediction models for either CL with/without palate or CPO, we extended the risk prediction analysis by explicitly considering all sub-phenotypes of oral clefts, defined by their clinical manifestations (e.g., the pattern of symptoms). By using a more refined sub-phenotype, it is possible to detect genetic variants contributing to a refined sub-phenotype, leading to an improved risk prediction model for the sub-phenotype. The details of analyses are summarized in **Table 2** and the ROC curves of risk prediction models for all sub-phenotype groups are depicted in **Figure 1** (CL/P) and **Figure 2** (CPO). Consistent with our current understanding of oral clefts, left CLP, right CLP and bilateral CLP showed similar genetic etiologies and were combined into one group by the MLRE method. The area under the ROC curve (AUC) of the CLP risk prediction model, which comprised of rs6072081 (*MAFB*), rs2073485 (*IRF6*) and rs560426 (*ABCA4*), was estimated to be 0.572 with 95% confidence interval (CI) of (0.556, 0.589). Left CL and right CL were also treated as a single entity by the MLRE method. The CL risk prediction model (**Figure 1**) with rs2073485 (*IRF6*) and rs590223 (*IRF6*) selected as risk predictors has an AUC value of 0.589 with 95% CI of (0.562, 0.617). Contrary to most of the current findings, our analyses treated cleft palate-soft only and cleft palate-hard as two separate groups. For these two groups, rs227731 (*17q22*), rs8610209 (*IRF6*), and rs2514527 (*GDF6*) were selected as risk predictors for cleft palate-soft only, whereas rs2073485 (*IRF6*), rs17389541 (*IRF6*), and rs1530300 (*8q24*) jointly contributed to the risk of cleft palate-hard. The risk prediction model formed for cleft palate-soft only (**Figure 2**) achieved an AUC value of 0.617 with 95% CI of (0.583,0.652), while the risk prediction model formed for cleft palate-hard (**Figure 2**) had an AUC value of 0.623 with 95% CI of (0.583,0.663).

**Table 2:** Risk prediction model for each sub-phenotype group of oral clefts.

Selected SNPs	Allele	Chromosome	Gene	Position	AUC
**Cleft lip and palate**					
rs6072081	A/G	20	*MAFB*	39261054	0.572(0.556,0.589)^a^
rs2073485	A/G	1	*IRF6*	209962794	
rs560426	A/G	1	*ABCA4*	94553438	
**Cleft lip only**					
rs2073485	A/G	1	*IRF6*	209962794	0.589(0.562,0.617)
rs590223	A/G	1	*IRF6*	209946707	
**Cleft palate – hard**					
rs2073485	A/G	1	*IRF6*	209962794	0.623(0.583,0.663)
rs17389541	A/G	1	*IRF6*	208053795	
rs1530300	C/T	8	*8q24*	129988640	
**Cleft palate-soft only**					
rs227731	A/C	17	*17q22*	54773238	0.617(0.583,0.652)
rs861020	A/G	1	*IRF6*	209989270	
rs2514527	A/C	8	*GDF6*	97169326	

*^a^95% confidence interval (CI).*

We also compared the risk prediction models formed by MLRE to those formed based on empirical knowledge. For such purposes, as suggested by previous literature, we classified the oral clefts into two sub-phenotype groups (i.e., CL/P and CPO). The same forward selection algorithm was then used to search for the most parsimonious risk prediction models for CL/P and CPO. The details of the risk prediction models are summarized in **Table 3** and the ROC curves for CL/P and CPO are depicted in **Figures 1** and **2**, respectively. The risk prediction model for CL/P, which comprised of rs2073485 (*IRF6*) and rs7078160 (*VAX1*), achieved an AUC value of 0.556 with 95% CI of (0.542, 0.570). For CPO, rs227731 (*17q22*), rs8610209 (*IRF6*), and rs2514527 (*GDF6*) were selected as risk predictors, and the corresponding AUC value of the model was 0.604 with 95% CI of (0.577, 0.631). While there is a considerable overlap of predictors selected by MLRE and the conventional approach, AUCs of the risk prediction models formed by the convention approach without considering phenotypic heterogeneity were smaller than those built by the MLRE method (**Figures 1** and **2**).

**Table 3:** Risk prediction models for cleft palate only and cleft lip with and without palate.

Selected SNPs	Allele	Chromosome	Gene	Position	AUC
**Cleft palate only**					
rs227731	A/C	17	*17q22*	54773238	0.604(0.577,0.631)^a^
rs861020	A/G	1	*IRF6*	209989270	
rs2514527	A/C	8	*GDF6*	97169326	
**Cleft lip with and without palate**					
rs2073485	A/G	1	*IRF6*	209962794	0.556(0.542,0.570)
rs7078160	A/G	10	*VAX1*	118827560	

*^a^95% CI.*

## Discussion

Genetic risk prediction studies have been recognized as an important step toward personalized genome medicine. Substantial evidences suggest that the oral clefts with the same or similar clinical manifestations have heterogeneous pathophysiological and etiological mechanisms (Harville et al., 2005; Mossey et al., 2009; Morris et al., 2010; Dixon et al., 2011). In the presence of phenotypic heterogeneity, methods treating all sub-phenotypes as the same outcome are subject to low predictive power, as the effects of genetic variants associated with one unique sub-phenotype are attenuated in the whole population (Mossey et al., 2009; Morris et al., 2010; Dixon et al., 2011). However, most of the genetic prediction studies of oral clefts do not explicitly explore the effect of phenotypic heterogeneity either because there is limited information to classify cases into homogeneous sub-phenotype groups or the sample size for each sub-phenotype is too small for valid statistical inference. Therefore, studies that infer homogeneous sub-phenotype groups based on empirical data could facilitate the identification of new genetic variants, leading to more accurate risk prediction models. In this study, we considered all sub-phenotypes of oral clefts and searched for genetic variants contributing to homogenous sub-phenotype groups of oral clefts. Among the sub-phenotypes considered, left CLP, right CLP, and bilateral CLP showed similar genetic etiologies, while left and right CL shared similar genetic causes. Different from traditional studies, where cleft palate-soft only and cleft palate-hard were treated as a single entity, our analysis suggested that they did not have exactly the same underlying genetic etiology. Although other criteria could be used to define the initial sub-phenotype groups, this study made use of the information from clinical diagnosis to define initial sub-phenotype groups. To the best of our knowledge, no prior risk prediction studies were conducted based on each sub-phenotype of oral clefts with the consideration of both phenotypic heterogeneity and gene-gene interactions. The approach used in this study could serve as the first step toward inferring homogeneous sub-phenotype groups of oral clefts, identifying new genetic variants associated with each sub-phenotype group, and exploring risk prediction models for each sub-phenotype group. With knowledge accumulated through further investigation and validation, a more accurate definition of oral clefts can be established, which might help us to better understand the process of the craniofacial anomalies in embryonic development and to build new diagnostic and therapeutic strategies to prevent the abnormity formed at embryonic stage.

Consistent with most of current findings, the interferon regulatory factor 6 (*IRF6*) has been selected as an important predictor for all forms of oral clefts (Zucchero et al., 2004; Ghassibe et al., 2005; Scapoli et al., 2005; Richardson et al., 2006; Park et al., 2007; Vieira et al., 2007a; Birnbaum et al., 2009; Grant et al., 2009; Beaty et al., 2010; Mangold et al., 2010). Starting from the 7th week of embryonic development, the palatal shelves rise to a horizontal position above the tongue and come into contact, and *IRF6* is one of the most important factors to ensure the palatal shelves rise and adhesive correctly (Sperber, 2002; Mossey et al., 2009; Dixon et al., 2011). *IRF6* transcription is activated by *p63*, which underlies several malformation syndromes including oral clefts (Sun et al., 2000; Ashique et al., 2002; Thomason et al., 2008; Mossey et al., 2009). The protein encoded by *IRF6* contribute to the development of van der Woude’s syndrome and popliteal pterygium syndrome, both of which are characterized by various degrees of cleft lip, cleft lip and palate, and CPO (Kondo et al., 2002; Richardson et al., 2006). In addition to *IRF6*, v-maf musculoaponeurotic fibrosarcoma oncogene homolog B (*MAFB*) and intron 6 of the ATP-binding cassette subfamily A member 4 (*ABCA4*) also contribute to the risk of cleft lip and palate (Mossey et al., 2009; Beaty et al., 2010; Huang et al., 2012). The genetic regions close to *MAFB* harbor numerous binding sites for transcription factors that are known to play a role in palate development (Beaty et al., 2010; Huang et al., 2012). Animal models further confirm the role of *MAFB* in oral clefts. In mouse, the *MAFB* mRNA and protein were highly expressed in the epithelium around the palatal shelves and in the medial edge epithelium during palatal fusion (Beaty et al., 2010). Although no evidence of *ABCA4* expression has been observed in mouse palatal shelves and no apparent relationship between *ABCA4* and oral clefts, *ABCA4* may be served as a surrogate to a nearby disease-associated gene (Beaty et al., 2010). Traditionally, cleft lip only and cleft lip with palate are treated as a single entity with homogenous genetic etiology while CPO has its own mechanism, as the cleft lip and primary palate have different developmental originals from the secondary palate (Fraser, 1955, 1970; Dixon et al., 2011). Recently, several studies suggest that the etiology of cleft lip may also be different from that of cleft lip with palate (Harville et al., 2005). Nevertheless, our analysis is not supportive of the completely unified genetic etiology of CL and CLP, although CL only and CLP do share some risk factors. It remains controversial whether cleft lip should be treated the same as cleft lip with palate due to the complex and heterogeneous nature of the disease.

It is noteworthy that based on our analyses cleft palate-hard and cleft palate-soft only should be treated as two separate sub-phenotypes of isolated cleft palate. Our results highlighted the potential that the hard and soft forms of isolated cleft palate have their own mechanisms each though they share some of the genetic risk factors. Treating all forms of isolated CPO as a single entity may jeopardize our ability to uncover important genetic risk predictor predisposing to a specific sub-phenotype. Nevertheless, current studies considered hard and soft cleft palate as variants of the same defect without systematic investigation on each of them. This is partially due to limited knowledge of disease etiology and relatively small sample size of sub-phenotypes of CPO. Our analyses could be served as an initial step toward exploring sub-phenotypes of CPO, and subsequent explorations and validations of etiologies underlying various sub-phenotypes hold great promise to move forward our understanding of cleft palate, which may eventually lead to new diagnostic and therapeutic strategies for CPO.

One possible limitation of the study is that only genes previously reported to be associated with oral clefts were included. Consequently, new genetic variants contributing to a specific sub-phenotype of oral clefts were not investigated in this study, which could lead to low performance of risk prediction models. A natural extension of current analysis is to conduct a genome-wide risk prediction analysis with the consideration of both gene–gene interactions and phenotypic heterogeneity. Another limitation of this study is related to the sub-phenotype definition. We used the sub-phenotypes defined in the data as our starting point and gradually combined the groups with similar genetic etiologies. The formed risk prediction models could have low performance if the initial sub-phenotype groups do not reflect the underlying genetic etiology. It is worthwhile to investigate other criteria of defining initial groups in future analysis.

## Conclusion

We used a newly developed statistical method to form risk prediction models for oral clefts with the consideration of heterogeneous disease etiology. The method first combined sub-phenotype groups shared similar genetic etiology, and then constructed risk prediction models for each newly formed sub-phenotype group. Further replication and follow-up studies are needed to validate the findings, but our analyses could serve as an initial step toward constructing risk prediction models for homogeneous sub-phenotype groups of oral clefts.

## Conflict of Interest Statement

The authors declare that the research was conducted in the absence of any commercial or financial relationships that could be construed as a potential conflict of interest.
